# The novel dynamic MPFL-reconstruction technique: cheaper and better?

**DOI:** 10.1007/s00402-021-04198-z

**Published:** 2021-10-11

**Authors:** Hauke Horstmann, Roman Karkosch, Annika Berg, Christoph Becher, Maximilian Petri, Tomas Smith

**Affiliations:** 1 Department of Orthopaedic Surgery, Diakovere Annastift, Hannover Medical School, Anna-von-Borries-Str. 1-7, 30625 Hannover, Germany; 2 Center for Hip, Knee and Foot Surgery, ATOS Clinic Heidelberg, 69115 Heidelberg, Germany

**Keywords:** Knee, MPFL reconstruction, Patella instability, Patella dislocation, Stabilization

## Abstract

**Purpose:**

Reconstruction of the medial patellofemoral ligament (MPFL) is an established procedure to restore patellar stability. Aim of this study is to evaluate the results of a dynamic MPFL reconstruction technique in a large university hospital setting.

**Methods:**

Two hundred and thirteen consecutive patients with 221 knees were surgically treated for recurrent lateral patellar dislocation. All patients obtained dynamic reconstruction of the MPFL with detachment of the gracilis tendon at the pes anserinus while maintaining the proximal origin at the gracilis muscle. Patellar fixation was performed by oblique transpatellar tunnel transfer. Follow-up data including Kujala and BANFF score, pain level as well as recurrent patella instability were collected at a minimum follow-up of 2 years.

**Results:**

Follow-up could be obtained from 158 patients (71%). The mean follow-up time was 5.4 years. Mean pain level was 1.9 ± 2.0 on the VAS. Mean Kujala score was 78.4 ± 15.5. Mean BANFF score was 62.4 ± 22.3. MPFL-reconstructions that were performed by surgeons with a routine of more than ten procedures had a significantly shorter surgical time 52.3 ± 17.6 min. Male patients yielded higher satisfaction rates and better clinical scores compared to females. Complications occurred in 27.2% of procedures, 20.9% requiring revision surgery of which were 9.5% related to recurrent patellar instability. 78% of all patients indicated they would undergo the procedure again.

**Conclusion:**

Dynamic MPFL reconstruction presents a reproducible procedure with increased complication rates, inferior to the results of static reconstruction described in the literature. Despite, it appears to be an efficient procedure to restore patellar stability in a large university hospital setting, without the necessity for intraoperative fluoroscopy.

**Trial registration:**

The study was registered in ClinicalTrials.gov with the registration number NCT04438109 on June 18th 2020.

## Introduction

Acute dislocation of the patella is a frequent injury of the knee joint with an incidence of 77 per 100,000 per year [27]. Recurrent dislocation occurs in up to 40% of cases after non-surgical management [5, 11]. While primary dislocations can be approached with conservative treatment, a surgical approach may be required in cases of recurrent dislocations [1]. The main goal of these operations is to avoid recurrent instability. In vivo, dynamic and static structures ensure a centered and stable patellar motion throughout extension and flexion of the knee joint. The medial patellofemoral ligament (MPFL) presents the primary restraint to lateral forces on the patella in extension [5]. The patella almost exclusively dislocates laterally [7]. It is commonly accepted that the MPFL is always impaired in the acute traumatic dislocation. To counteract the lateral patella glide, MPFL reconstruction is favored by several authors and has proven to restore stability [6, 29]. When excluding other anatomic risk factors for patellar instability, such as trochlea dysplasia, patella alta, genu valgum, and muscular hypotrophy, isolated reconstruction of the MPFL can restore stability of the patella during motion of the knee joint and nowadays represents the primary treatment for recurrent patellar instability [8, 25]. To date, several grafts and techniques have been reported for MPFL reconstruction.

Most commonly, static reconstruction using a hamstring tendon or partial-thickness quadriceps graft is performed with femoral interference screw fixation. Patellar fixation is usually either performed with suture anchors or interference screw. Complication rates of this static technique are reported up to 26.1% [26]. Despite the use of intraoperative fluoroscopy, most common problems include malpositioning of the femoral tunnel, resulting in either insufficiency of the graft with recurrent patellar instability, proudness of interference screw, osteolysis problems when using resorbable screws, weak fixation strength when screw sits too deep, malposition of cortical fixation (if cortical button fixation is chosen at the far cortex) or overtensioning of the graft with stiffness of the knee.

In an effort to avoid these femoral fixation issues and intraoperative fluoroscopy, a dynamic reconstruction technique has been developed. First described in 1904, the technique was enhanced and published by Ostermeier 2007 [21] and Becher 2014 [4]. This technique consists of detachment of the semitendinosus or gracilis tendon at the pes anserinus while maintaining the proximal origin at the muscle, avoiding the need for femoral graft fixation.

The purpose of this single-center retrospective study of consecutive patients was to evaluate the outcomes and complication rates for this dynamic MPFL-reconstruction technique in a large university setting.

## Materials and methods

### Participants

The study was approved by the local ethics committee. Written informed consent was obtained from each participant. Patients were identified through the hospitals database searching for MPFL reconstruction. The included surgical procedures were performed from 07/2010 to 12/2016 in a single orthopedic hospital. 15 orthopedic surgeons were involved. Five surgeons were performing 81.6% of the procedures. Inclusion and exclusion criteria are displayed in Table 1.

**Table 1:** Display of inclusion and exclusion criteria

Inclusion criteria:
Dynamic MPFL reconstruction of recurrent patellofemoral instability (primary and revision surgery)
Age of 18 years or older at a minimum of 24-month follow-up
Exclusion criteria:
Diagnosis of connective tissue disease
Neurological diseases
Concomitant alignment correction surgery including femoral/tibial osteotomy
Trochleoplasty and tibial tubercle transfer

Two hundred and thirteen patients with 221 knees (8 bilateral) met the inclusion criteria. All patients were contacted by mail or phone to independently answer questionnaires. Questionnaires for 158 knees were returned (71.5%). Among the patients lost to follow-up, 47 could not be reached either by mail or phone, 14 refused to participate.

### Surgical technique

**Fig. 1:**
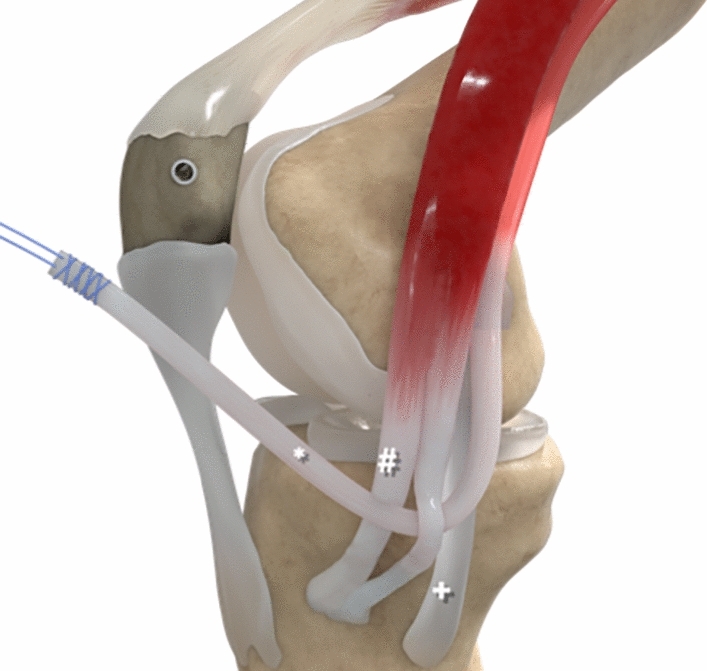
Surgical technique; * gracilis tendon, # sartorius fascia, + medial collateral ligament

**Fig. 2:**
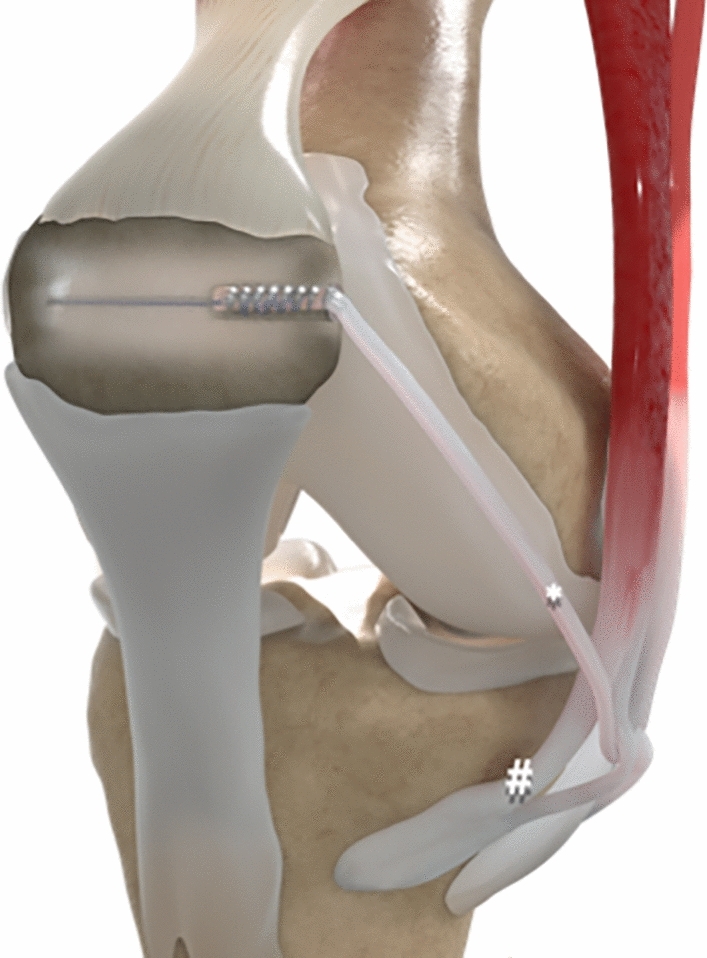
Surgical technique; * gracilis tendon, # sartorius fascia, + medial collateral ligament

The technique was performed similar to the description by Becher et al. [4]. In contrast to the original technique, the gracilis tendon was passed around the incised sartorius fascia and shuttled subcutaneously to the medial patellar margin, allowing the sartorius fascia to act as a pulley for the transferred tendon (Figs. 1, 2).

### Clinical outcome measures

Data collection was performed by self-administered questionnaires, which were sent to the patients.

The medical records and radiographs of all patients were reviewed for demographic information (age, sex) and to identify complications related to the surgical procedure.

Rating of the results was performed using the Kujala score and the BANFF score 2.0. The Kujala score is a patient-reported assessment. It contains 13 questions that add up to a total score of 100 points. The worst value is zero points [14]. The BANFF score 2.0 (or BANFF patellar instability instrument (BPII) 2.0) is a patient-reported quality of life assessment. It consists of 23 items that add up to one score. The range of possible values is from 0 to 100 with 100 as the best result [3, 10].

Pain level was recorded using the visual analogue scale (VAS, 0 = no pain, 10 = severe pain). General satisfaction with treatment outcomes was evaluated by questionnaire (1 = excellent, 2 = very good, 3 = good, 4 = fair, 5 = poor, 6 = very poor) and by asking patients whether they would undergo the procedure again. Furthermore, information on patient characteristics, recurrence of dislocation, revision surgery, and other postoperative complications were recorded.

Postoperative X-rays were analyzed concerning intraoperative complications (e.g., fractures or patellar drill hole malpositioning).

### Postoperative care

After the surgical procedure, a brace was applied for 2 weeks 24/7 plus 2 more weeks only at night. In the hospital phase, physiotherapy was performed once a day. During exercises, the brace was removed. Weight bearing was limited for 3 weeks past operation. Full weight bearing was applied after 3 weeks. Patients received standard physiotherapist-supervised rehabilitation protocol for 2 months.

### Statistics

Descriptive analysis was performed using means and standard deviation. Subgroups were compared using a 2-tailed Student *t* test for normal distribution, two-tailed Fishers exact test and the χ2-test. A two-sided *P* value of 0.05 was considered to indicate statistical significance. All reported *P* values are two-sided and were not adjusted for multiple comparisons. Statistical comparisons were made utilizing SPSS software (SPSS, Chicago, Illinois), version 24. Pearson correlation was calculated. Missing data were not included in the statistical analyses.

## Results

Patient characteristics are reported in Table 2. Among 158 MPFL-reconstructions, six patients had bilateral MPFL-reconstructions. The mean age at the time of procedure was 22.5 ± 8.1 with a mean follow-up time of 5.4 ± 1.8 years. The mean time span of the procedure was 54.1 ± 18.3 min. 54 patients underwent concomitant procedures such as cartilage repair (*n* = 34), loose body excision (*n* = 19), proximal soft tissue realignment (Insall) (*n* = 10), lateral release (*n* = 7), meniscal repair (*n* = 1), and lateral patellar facetectomy (*n* = 2).

**Table 2:** Patient characteristics

	*n*	Age at time of procedure (years)	Side of operation (left/right)	Follow-up time (years)	Time of procedure (min)	Previous operation (no/yes)
Female	106	21.8 ± 8.1	66/40	5.5 ± 1.8	52 ± 16	75/31
Male	52	24.1 ± 7.9	30/22	5.2 ± 2.0	58 ± 21	37/15
Overall	158	22.5 ± 8.1	96/62	5.4 ± 1.8	54 ± 18	109/49

The main outcome parameters are shown in Tables 3 and 4. Male patients showed significantly better clinical outcome scores (VAS, Kujala and BANFF score, satisfaction) compared to female patients. Patients of 123 knees (78%) indicated they would undergo the procedure again. 75% of the female patients and 83% of the male patients would repeat the operation. The cohort returned to their sport at preoperative levels after a mean of 9.1 (± 3.7) months. Female patients returned to their sport slightly later with 9.3 ± 3.7 months, while male patients exercised in their preoperatively sport at 8.6 ± 3.7 months.

**Table 3:** Outcome measures at latest available follow-up; values are expressed as mean ± standard deviation, * indicates statistically significant

	VAS pain	Kujala score	BANFF score	Satisfaction
Female	2.3 ± 2.2*	75 ± 16*	58 ± 23*	2.8 ± 1.2*
Male	1.0 ± 1.4*	85 ± 11*	72 ± 18*	2.0 ± 1.1*
Overall	1.9 ± 2.0	78 ± 16	62 ± 22	2.5 ± 1.2

**Table 4:** Procedure-related complications; single operations with multiple procedures possible

Procedure-related complications	Treatment	Quantity
Cases with recurrent instability		*n* = 23 (14.6%)
Recurrent instability	Insall proximal realignment	*n* = 4 (3%)
	Revision MPFL reconstruction	*n* = 9 (6%)
	Insufficient preoperative diagnosis with relevant concomitant pathologies	
	-Distal femoral osteotomy	*n* = 2 (1%)
	-Trochleoplasty	*n* = 2 (1%)
	-Tibial tubercle osteotomy	*n* = 1 (1%)
Stiffness	Manipulation under anesthesia	*n* = 1 (1%)
Singular traumatic dislocation	Conservative treatment	*n* = 8 (5%)

When comparing the patients who got isolated MPFL reconstruction to the patients that got MPFL reconstruction and additional concomitant procedure no statistically significant difference in the Kujala and BANFF Score as well as the return to sport time and VAS was observed.

### Radiographic findings

No patella fracture was observed. The drill hole position was placed at 47.3% ± 8.9% of the total patella length in cranio-caudal direction. No significant correlation could be found between drill hole position in cranio-caudal direction and VAS, nor Kujala score or BANFF score.

### Complications

At an average follow-up of over 5 years, a total of 43 (27%) complications occurred, of which were 27 (17%) procedure related (Table 4) and 16 (10%) were not procedure related (Table 5).

**Table 5:** Not procedure-related complications

Not procedure-related complications	Treatment	Quantity
Hematoma	Hematoma drained	*n* = 2 (1%)
Infection	Debridement	*n* = 2 (1%)
Suture granuloma	Revision	*n* = 1 (1%)
Recurrent cartilage defect	Hyaluronic scaffold with bone marrow stimulation	*n* = 1 (1%)
Progredient osteoarthritis	Lat. retinacular release, chondroplasty, synovectomy	*n* = 5 (3%)
Hypertrophic scar	Release of MPFL reconstruction	*n* = 1 (1%)
Deep-vein thrombosis	Sufficient medical treatment	*n* = 1 (1%)
Unknown complication	Unknown procedure	*n* = 3 (2%)

## Discussion

The most important finding of this study was that dynamic MPFL reconstruction is able to restore patellar stability with good patient satisfaction rates but shows higher complication rates and inferior outcomes in Kujala and BANFF score compared to static MPFL reconstruction [24, 26].

Static techniques of MPFL reconstruction are well examined and have proven to restore patellar stability [8]. Several authors reported a postoperative increase in the Kujala score [8, 19, 29]. Mean Kujala score in our examination was 75.5 in females and 84.8 in males. Enderlein et al. found a Kujala score of 77 in their large follow-up study of 224 patients at a mean follow-up of 41 months [8]. Schneider et al. reported a higher mean Kujala score of 85 in their meta-analysis of static MPFL reconstruction [24]. Our findings are in the lower region of previously published data, which presents with Kujala scores that range from 74.7 to 94.5 [2, 12, 13, 15, 16, 18, 22–24, 26]. Becher et al. compared clinical and radiological outcomes of static and dynamic medial patellofemoral ligament (MPFL) reconstruction techniques in a retrospective matched-pair cohort of 30 patients. They found no significant differences in mean Kujala, Tegner, Lysholm, or visual analogue scale scores or radiographic parameters, with high satisfaction rates in both groups. To best of our knowledge, this is the only study to date to compare results of static and dynamic MPFL reconstruction [4]. According to Shah et al., reported complication rates in isolated MPFL reconstruction vary tremendously ranging between 0 and 85.2% [17, 20]. In their literature review, they found a mean complication rate of 25.7 ± 21.3% across all included studies [26]. The rate of postoperative recurrent patellar instability ranges from 0 to 32.7% [9, 24]. Yet, some authors describe significantly lower re-dislocation (1.2%; 4%) and re-operation rates (3.1%) [24, 26]. Compared to these reported rates, our overall complication rate of 27.2% and postoperative recurrent patellar instability rate of 14.6% are highly increased. We believe that this is due to “undertreatment” with MPFL reconstruction as a standalone procedure in our cohort, especially during the early phase of recruitment. Malalignment and hyperlaxity account for a higher risk for patellar dislocation [24, 26]. Anatomic risk factors for patellar instability include trochlea dysplasia, patella alta et lateralisata, genu valgum, and muscular hypotrophy. It is paramount to properly identify these risk factors prior to procedure planning. According to the “à la carte” concept, surgical procedure needs to address these risk factors to reduce the risk of recurrent instability. Additional procedures such as trochleoplasty, valgus correction osteotomy, and tibial tubercle osteotomy might be required. It is our belief that rates of recurrent instability can be further decreased when stratification for accompanying risk factors is thoroughly performed in all cases.

There are some apparent differences when comparing data of male and female participants in this study. Men achieved significantly better results in all outcome scores (Kujala and BANFF) and reduced VAS values (1.0 ± 1.4 vs. 2.3 ± 2.2). Possible explanation for this finding includes the high prevalence of patellofemoral pain in females (annual prevalence: female 29.2% vs. male 15.5%) [28].

Reported return-to-sports rates in the literature range from 69 to 84% [15, 24]. In a study cohort of 39 competitive athletes undergoing MPFL reconstruction, return-to-sports rate was 85% at a mean of 8.1 months [13]. In our cohort, the return-to-sports rate was lower than in the literature, which might be due to a more heterogeneous cohort including patients with less active lifestyles. Furthermore, we did not collect any preoperative baseline data regarding sports activity levels of the patients. Therefore, we might have missed to preclude patients without prior sports activities from our analysis, potentially resulting in a falsely low rate of return-to-sports in our cohort. The mean time for return-to-sports of 9.1 months in our cohort is similar compared to previously reported numbers of return-to-sports following MPFL reconstruction [13].

Regarding the radiographic findings of patellar tunnel placement, no predictive value to clinical outcomes could be observed. It is crucial to avoid damaging the articular cartilage while drilling the tunnel. An oblique drilling direction is recommended in an effort to minimize the risk of patellar fracture, which represents a devastating complication in MPFL reconstruction. Of all complications, patellar fractures in MPFL reconstruction occur in up to 2.4% [26]. In our cohort, no patellar fracture was observed.

The drawbacks of static MPFL reconstruction include the need of intraoperative fluoroscopy to properly choose the anchoring point of femoral fixation of the graft. This requires a more elaborate setup and potentially prolongs the surgical time. Even when using fluoroscopy, picking the anatomic site of the femoral fixation remains the most common source of complications [22].

Therefore, a dynamic technique without detaching the tendon proximally might avoid these problems. Of note, in dynamic MPFL reconstruction, no intraoperative fluoroscopy is necessary.

Finally, by avoiding the need for intraoperative fluoroscopy and fixation on the femoral side, the dynamic fixation technique appears promising regarding reduction of radiation exposure on patient and surgeon side. Whether costs can be reduced as well has to be examined in further studies.

Several limitations apply to this study. First, no baseline scores preoperatively were collected. Second, with dynamic reconstruction being the standard therapy in our institution, no control group was established. Third, the study cohort is heterogeneous in terms of concomitant pathologies. This particularly applies to the earlier phase of this study cohort, when the “à la carte” operation to the patella was not fully established yet. Fourth, we did neither discriminate between the prior athlete level in patients nor did we determine whether the sports level returned to was equal to the prior level.

## Conclusion

Dynamic MPFL reconstruction presents a reproducible procedure with increased complication rates, inferior to the results of static reconstruction described in the literature. Despite, it appears to be an efficient procedure to restore patellar stability in a large university hospital setting, without the necessity for intraoperative fluoroscopy and femoral side fixation devices.

## Data Availability

The data that support the findings of this study are available from the corresponding author, Horstmann H, upon reasonable request.

## Abbreviations

MPFL: Medial patellofemoral ligament
VAS: Visual analog scale
OPS-codes: Codes of surgical procedures
